# Analysis of radical scavenging active components in the fermented mycelia of *Ophiocordyceps formosana*

**DOI:** 10.1080/21501203.2017.1383318

**Published:** 2017-10-10

**Authors:** Yang-mei Li, Wen-ming Cheng, Zi-fei Da, Fenglin Hu, Chun-ru Li

**Affiliations:** a Anhui Provincial Key Laboratory of Microbial Pest Control, Anhui Agricultural University, Hefei, China; b School of Pharmacy, Anhui Medical University, Hefei, China; c Microbiology and Food Research Center, Zhejiang BioAsia Institute of Life Sciences, Pinghu, China

**Keywords:** *Hirsutella huangshanensis*, DPPH-free radical, hydroxyl radical, activity determination, LC–MS

## Abstract

The rates of 1,1-diphenyl-2-picryhydrazyl (DPPH)-free radical scavenging and hydroxyl radical eliminating were employed as indexes to qualitatively and quantitatively analyse the radical scavenging activity of the extracts with different solvents from fermented mycelia of *Hirsutella huangshanensis* RCEF0868, the anamorph of *Ophiocordyceps formosana* (FMOF). The results showed that both the aqueous extract and the methanol extract had significant radical scavenging activity. The half maximal inhibitory concentrations (IC_50_) of the aqueous extract on DPPH-free radical and hydroxyl radical are 0.85 and 1.37 mg/mL, respectively, while the IC_50_ of methanol extract is 1.23 and 2.91 mg/mL, respectively. The combined liquid chromatography-diode array detector-high resolution mass spectrometer(HRMS) analysis and activity determination revealed that the molecular formulas of three radical scavengers in aqueous extract were C_6_H_14_O_6_(1), C_14_H_17_N_5_O_8_(2) and C_20_H_33_N_5_O_9_(3). In addition to the compound 1, the methanol extract had another three radical scavengers as C_30_H_22_O_11_(4), C_30_H_22_O_10_(5) and C_30_H_18_O_10_(6). All the six kinds of components exhibited the DPPH radical scavenging activity, and compounds 1, 4 and 5 also showed the hydroxyl radical eliminating activity at the same time. By querying the database of natural products and reference to the relevant reports, compound 3 may be a new entity. The others were respectively cordycepic acid, succinoadenosine, 4α-oxyrugulosin, rugulosin and skyrin and, among them, succinoadenosine and 4α-oxyrugulosin were found in entomogenous fungi for the first time.

## 1. Introduction

*Ophiocordyceps formosana* (Kobayasi & Shimizu) C. R. Li, M. Z. Fan and Z. Z. Li was first discovered in 1981 from Taiwan. It was also found in Huangshan Mountain of Anhui Province in mainland China (Li et al. 2002, 2014), and *Hirsutella huangshanensis* was identified as its anamorph (Li et al. 2005).

Subsequently, our group also reported the culture technology of mycelia and fruiting body of *O. formosana* (Li et al. 2005, 2011), together with part of the active ingredients research (Li et al. 2014). For instance, the composition and content of volatile components of mycelia were significantly affected by different culture ways of *O. formosana* (Ding et al. 2012). Carbonyl amino acids, cyclic peptide antibiotics etc. were detected in the fruiting body of *O. formosana* (He et al. 2007, 2010). Also, some reports on the comparison of cytotoxic extracts from fruiting bodies, infected insects and cultured mycelia of *O. formosana* revealed that rugulosin was cytotoxic against Chinese hamster ovary (CHO) cells, while skyrin was non-cytotoxic against CHO cells (He et al. 2007, 2008; Lu et al. 2014).

Free radicals were regarded as harmful compounds produced by organism oxidation reaction, due to its unpaired electron and reactive oxygen causing oxidative damage to DNA, lipid, protein and other biological molecules when the endogenous antioxidant defence was insufficient (Aruoma 1998). For example, increase in the formation or metabolic loss of free radicals was closely related to most of the cardiovascular diseases. Studies have reported that preventing the uncoupling of vascular endothelial nitric oxide synthase brought a new way to the treatment of these diseases (Chen et al. 2012). However, the free radical scavenging activity and its chemical base of *O. formosana* have not yet been published. This article did some comparative study on 1,1-diphenyl-2-picryhydrazyl (DPPH)-free radical and hydroxyl-free radical scavenging activity of water extract and methanol extract of *H. huangshanensis* and analysed their bioactive constituents by liquid chromatography–mass spectrometry (LC–MS), which accumulated data for the development of biologically active ingredients in the insect fungus.

## 2. Materials and methods

### 2.1. Strain

*H*. *huangshanensis* RCEF0868 was provided by the Research Center on Entomogenous Fungi in Anhui Agricultural University and also kept in China General Microbiological Culture Collection Center (CGMCC No. 4147). Fermented mycelia of *Ophiocordyceps sinensis* (FMOS) was purchased from Zhejiang Hangzhou Xueyu Biotech Co., Ltd. (Batch no. 201201215).

### 2.2. Equipment

HZQ-F160 full temperature shock incubator, Harbin East Electronics Company; FreeZone12 freeze-drying system, the United States Labonconco Company; KQ5 200DE CNC ultrasonic cleaner, Kunshan Ultrasonic Instrument Co., Ltd.; 2K-15 ultracentrifuge, Sigma Company; SpectraMaxM2 microplate reader, USA Molecular Device Company; centrifugal vacuum concentrator, Germany Christ Company; resolution LC–MS analyser (Agilent 6210 time-of-flight [TOF] LC/MS), including the Agilent 1100 high-performance liquid chromatography (HPLC), diode array detector (DAD), high-performance TOF MS with an electrospray ionisation (ESI) source and an Agilent workstation, Agilent; analytical separations was Phenomenex Synergi Hydro-RP (4 μm, 4.60 × 250 mm).

### 2.3. Chemicals

HPLC grade methanol offered by Shanghai Xingke Biochemical Co., Ltd.; HPLC grade acetonitrile, Tedia Company; DPPH, Sigma Company; other reagents were analytical grade chemicals.

### 2.4. Shake culture

Each 250-mL flask with 100 mL of liquid special media was inoculated with mycelia mat (ca. 10 cm^2^) from a plate culture and incubated on a shaker at 130 rpm for 12 days at 25°C. The culture medium contained potato glucose medium complemented with maltose (1%), peptone (1%), KH_2_PO_4_ (0.3%), MgSO_4_ (0.15%), ammonium citrate tribasic (0.04%) and vitamin B (0.4%). The initial pH was adjusted to 6.5 before sterilisation.

### 2.5. Submerged culture

The strain was cultivated in a 30–300-L airlift fermentation system with working volume of 21 L, with inocula 8%, incubated at 25°C for 15 days under the following conditions: pressure 0.13 MPa, temperature 25 ± 1°C, the initial pH 6.5 and the air ventilation 1/0.8 (v/v). The media contained sugar (2%), milk powder (3%), yeast extract powder (1.5%), silk worm pupa powder (3%), ammonium citrate (0.04%), MgSO_4_ (0.15%) and KH_2_PO_4_ (0.3%). Mycelia were collected by centrifugation at 15,000*g* for 10 min after fermentation, washed for three times with sterile water. The mycelia were subjected to freeze drying and ground. All samples were stored at −20°C.

### 2.6. Extraction

Seven mycelia samples were weighed at a mass to liquid ratio of 30 mg/mL and respectively extracted with water, methanol, ethanol, acetone, ethyl acetate, *n*-butanol and petroleum ether at 30°C, 80 kHz ultrasound for 30 min and then kept at 4°C overnight. The supernatant was measured after centrifugation.

### 2.7. Bioassay methods

#### 2.7.1. DPPH-free radical scavenging assay

The mycelial extract solution (100 μL) and an equal volume of 0.2 mmol/L DPPH ethanol solution were added to the 96-well microtitre plate, shock mixed for 30 s and then left in the darkness at room temperature for 20 min. The absorbance was measured at 517 nm (*A*_1_). The absorbance of solvent instead of mycelium extract solution was measured as *A*_0_ and that of sample solution without DPPH solution measured as *A*_2_ (Liu et al. 2010). Each sample was determined in triplicate. The scavenging rate of DPPH radicals by the sample was calculated according to the following equation:

(1) $$ScavengingrateofDPPHradicals\left(\%\right)=\left[1-\frac{\left(A_{1}-A_{2}\right)}{A_{0}}\right]\times 100\%.$$

#### 2.7.2. Hydroxyl radical scavenging assay

Ninety six-well microtitre plate was successively added with 50 μL 6 mmol/L FeSO_4_ solution, 50 μL mycelium extract solution and 50 μL 6 mmol/L salicylic acid ethanol solution. After vortex mixing, 50 μL 6 mmol/L H_2_O_2_ was added to initiate the reaction, and then left at room temperature for 20 min after vortex mixing. The absorbance was measured at 510 nm (*A*_1_). The absorbance of the solvent in place of the mycelial extract solution was measured as *A*_0_ and that of sample solution without H_2_O_2_ measured as *A*_2_, and each sample was tested triplicately. The equation for scavenging rate of hydroxyl radical by the sample was the same as Equation (1).

### 2.8. Separation by HPLC and analysis by HPLC-ESI-TOF-MS

The mycelial extract solution was chromatographed, and each tube was collected every minute. The separated components were concentrated and dried in the rotary apparatus, and then each aliquot was redissolved by adding 300 μL of extracting solvent. The bioassay of different components collected was performed.

HPLC conditions: injection volume of 20 μL, flow rate 0.8 mL/min, column oven temperature at 30°C; mobile phase: acetonitrile and 0.1% formic acid aqeous solution; elution gradient for the water extract: 0–10 min acetonitrile from 0% up to 5%, 10–35 min acetonitrile from 5% to 100%, 35–45 min to 100% acetonitrile elution 10 min; elution gradient for ethanol extract: 0–10 min from 0% to rise to 5%; 10–30 min acetonitrile from 5% to 100%, 30–45 min 100% acetonitrile elution 15 min. The range of detection wavelength was from 190 to 800 nm.

MS conditions: atomisation air pressure ESI ion source is 0.24 MPa (35 psi), a nitrogen flow rate of 12 L/min, capillary temperature was 325°C, ion scan range (*m*/*z*) 50–1000, the cationic ion mode of voltage 4000 V, debris voltage 250 V; ionisation voltage of 3500 V, debris voltage 175 V under negative ion mode.

## 3. Results and analysis

### 3.1. Determination of activity of different solvent extracts

**Figure 1.**
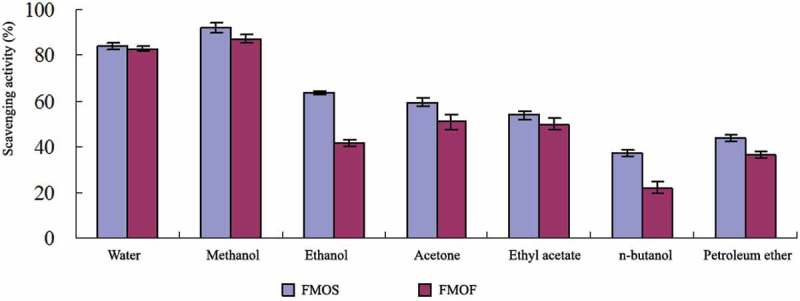
DPPH-free radical scavenging activity of different solvent extracts.

**Figure 2.**
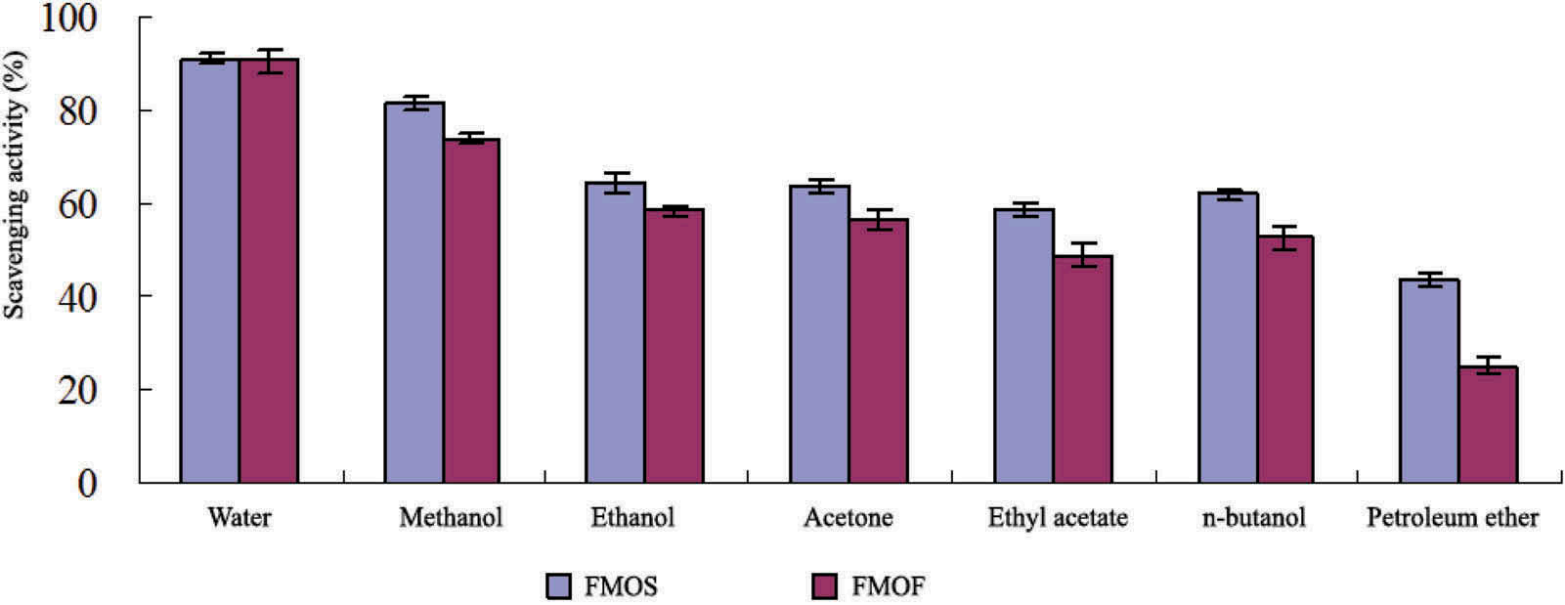
Hydroxyl radical scavenging activity of different solvent extracts.

The FMOS was used as the control, and FMOF was obtained according to the method described in Section 2.6 Activity assays of various solvent extracts were tested according to the method of Section 2.7, and the results are shown in Figures 1 and 2.

The results from Figures 1 and 2 showed that DPPH-free radical scavenging activity of the methanol extract is slightly potent, and the hydroxyl radical scavenging activity of the water extract is relatively strong. There was no significant difference in the scavenging-free radical activity of methanol and water extracts from FMOF compared with FMOS group.

The contents of free radical scavenging activity in the mycelia of RCEF0868 were soluble in both water and methanol. According to the principle of similar compatibility, it was deduced that the active component was dominated by high polarity and medium polarity. Compared with the FMOS, the scavenging activities of DPPH-free radicals and hydroxyl radicals in the two extracts of FMOF were similar, which indicated that FMOF had high application value on the antioxidant activity. It could be used as a suitable substitute for *O*. *sinensis*.

### 3.2. Free radical scavenging activity of the water and methanol extracts of FMOF

The sample solution of the aqueous extract and the methanol extract at a concentration of 5.0 mg/mL was precisely arranged according to the method described in Section 2.6 and then subjected to activity measurement according to the method described in Section 2.7 after dilution to different concentrations. The results are shown in Table 1.

**Table 1.** Free radical scavenging rates of crude extracts from different concentrations of FMOF.

	DPPH scavenging rates (%)		Hydroxyl radical scavenging rate (%)	
Concentration (mg/mL)	Water extract	Methanol extract	Water extract	Methanol extract
0.5	35.42 ± 1.23*	26.35 ± 0.70*	25.30 ± 0.49*	18.05 ± 1.16*
1.0	56.50 ± 1.84*	43.00 ± 1.31*	37.79 ± 1.15*	29.00 ± 1.01*
2.0	70.58 ± 0.96*	61.75 ± 1.27*	53.16 ± 0.69*	43.51 ± 1.64*
3.0	78.06 ± 0.63*	68.85 ± 1.44*	68.08 ± 1.71*	51.35 ± 1.20*
4.0	82.49 ± 1.45*	80.51 ± 1.05*	81.15 ± 1.69*	54.83 ± 1.09*
5.0	87.83 ± 1.61*	91.70 ± 0.84	90.04 ± 0.91	61.60 ± 0.62*
Vc	93.59 ± 0.39	93.84 ± 0.91	91.18 ± 0.30	91.34 ± 0.69

Vc: Vitamin C, 1.0 mg/mL. *Compared to Vc group.

The scavenging activity on DPPH and hydroxyl radicals of FMOF samples showed a significant concentration-dependent manner, and there was no significant difference in the scavenging-free radical activity between the high concentration and the Vc group (Table 1). With the increase of sample concentration, the scavenging activities of both free radicals were enhanced. The water extract of FMOF had obvious scavenging activity against DPPH and hydroxyl radicals, and the IC_50_ was 0.85 and 1.37 mg/mL, respectively. The clearance rate of the water extract at the concentration of 5.0 mg/mL reached 87.83% and 90.04% for DPPH and hydroxyl radicals, respectively, after reaction for 3 min. The scavenging activity on DPPH-free radicals of the methanol extract was slightly higher than that of the water extract, whereas the scavenging activity on hydroxyl radical of the water extract was obviously stronger than that of the methanol extract. The water extract of FMOF exhibited IC_50_ at 1.23 and 2.91 mg/mL, respectively. The clearance rate of the methanol extract at the concentration of 5.0 mg/mL was 91.70% and 61.60% for DPPH and hydroxyl radicals, respectively, after the reaction time.

### 3.3. HPLC–HRMS combined qualitative analysis of the active extract

#### 3.3.1. Analysis of active components by LC-DAD

**Figure 3.**
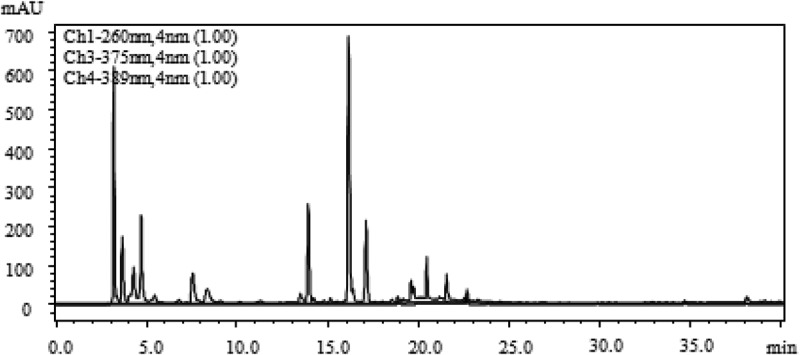
HPLC chromatogram of water extract of FMOF.

**Figure 4.**
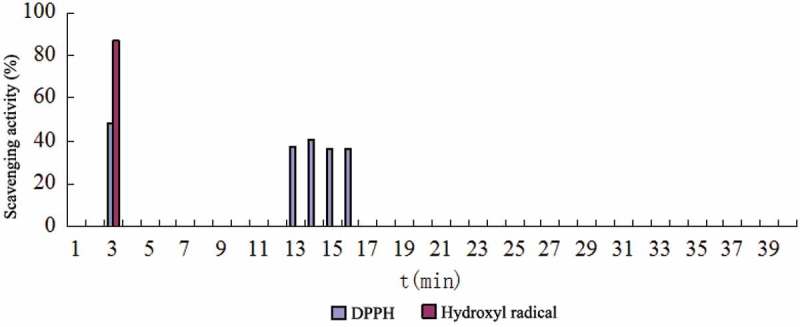
The free radical scavenging activity of the HPLC component of water extract from FMOS.

**Figure 5.**
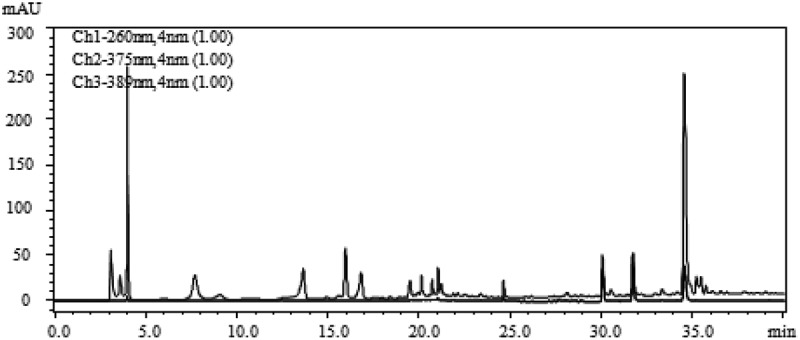
HPLC chromatogram of methanol extract of FMOF.

**Figure 6.**
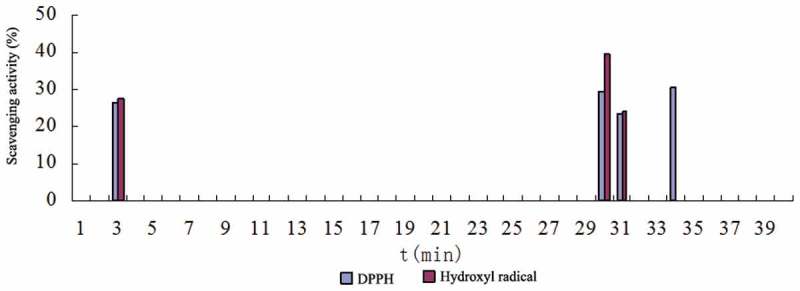
The free radical scavenging activity of the HPLC component of methanol extract from FMOS.

According to the method 2.8, the water extract and the methanol extract of FMOF were separated by HPLC, and free radical scavenging activity of each component was determined. The high-performance liquid chromatogram and activity determination results are shown in Figures 3–6.

There are several components in the water extract and the methanol extract of the FMOF, respectively, which have free radical scavenging activity (Figures 4 and 6). Among them, the active fractions of the water extract are mainly distributed in two parts, corresponding to the three chromatographic peaks at the retention time of 3.1, 13.9 and 16.1 min, respectively. The active parts of the methanol extract contained three chromatographic peaks at the retention times of 30.1, 31.7 and 34.6 min, respectively, besides the same peak as that of the water extract at the retention time of 3.1 min. The results showed that all the active components showed scavenging activity of DPPH-free radicals. Among them, three eluents at the retention time of 3.1, 30.1 and 31.7 min, respectively, showed both DPPH and hydroxyl radical scavenging activity.

#### 3.3.2. Qualitative analysis of active components by LC–MS

##### 3.3.2.1. Analysis of the active components of water extract by HPLC-TOF-MS

**Figure 7.**
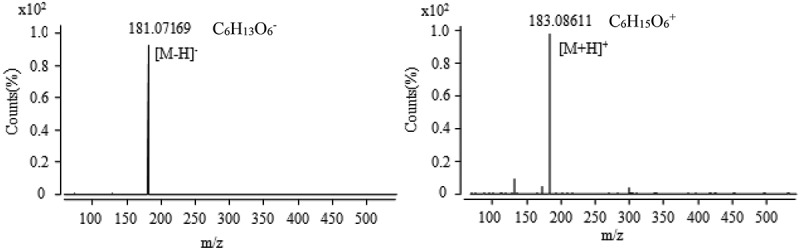
The anion and cation mass spectra of component at the retention time of 3.1 min.

The active fraction collected in the aqueous extract of Figure 4 for 3 min, including two chromatographic peaks in Figure 3, was recollected according to the peak, and the activity was determined to confirm the active fraction as a chromatographic peak with a retention time of 3.1 min. The active fraction was determined as the chromatographic peak with the retention time of 3.1 min. The active constituent was measured by the mass spectrometry (MS) method of Section 2.8 and the obtained mass spectrum was shown in Figure 7. It was found that there was a single mass-to-charge ratio (*m*/*z*) of 181.07169 (the relative error of 0.38 ppm) in the anionic mode, it can be seen that it is an excimer ion peak of [M − H]^−^ according to the ESI characteristic, the corresponding ionic formula is C_6_H_13_O_6_^−^. In the cationic mode, there is a peak with a mass-to-charge ratio (*m*/*z*) of 183.08611 (the relative error of 1.04 ppm), which is an excimer ion peak of [M + H]^+^, the corresponding ionic formula was C_6_H_15_O_6_^+^. According to the anion and cations analysis, the molecular formula of the component is C_6_H_14_O_6_. In combination with the natural products of related entomopathogenic fungi, and query the Chapman & Hall natural product database, it could be deduced that the active component is cordycepic acid (d-mannitol) shown in Figure 13.

**Figure 8.**
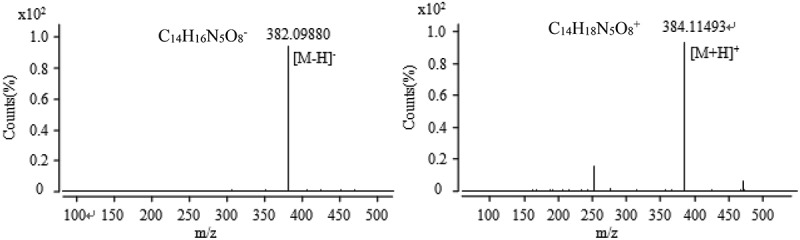
The anion and cation mass spectra of component at the retention time of 13.9 min.

**Figure 9.**
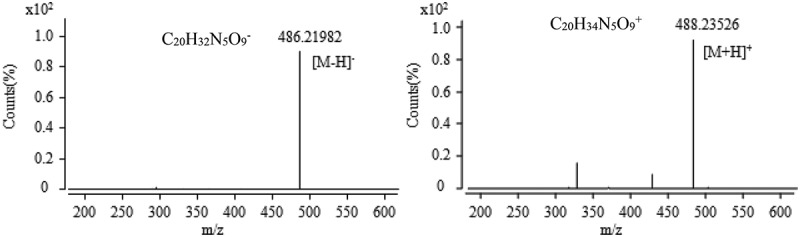
The anion and cation mass spectra of component at the retention time of 16.1 min.

**Figure 10.**
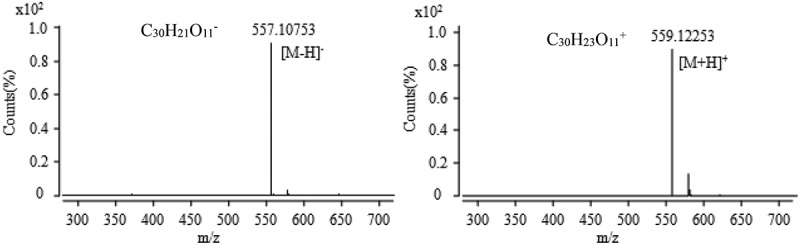
The anion and cation mass spectra of component at the retention time of 30.1 min.

**Figure 11.**
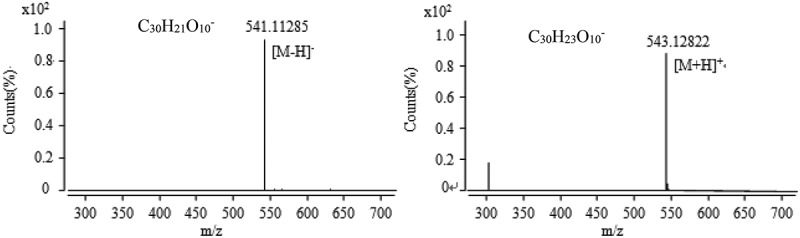
The anion and cation mass spectra of component at the retention time of 31.7 min.

**Figure 12.**
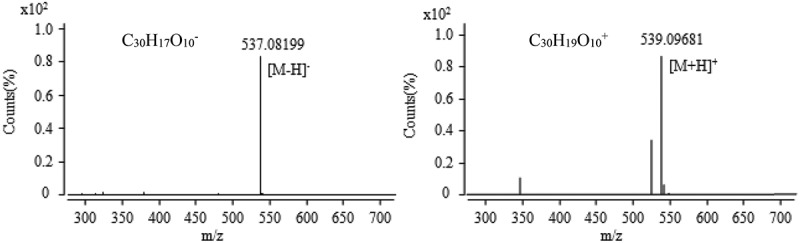
The anion and cation mass spectra of component at the retention time of 34.6 min.

**Figure 13.**
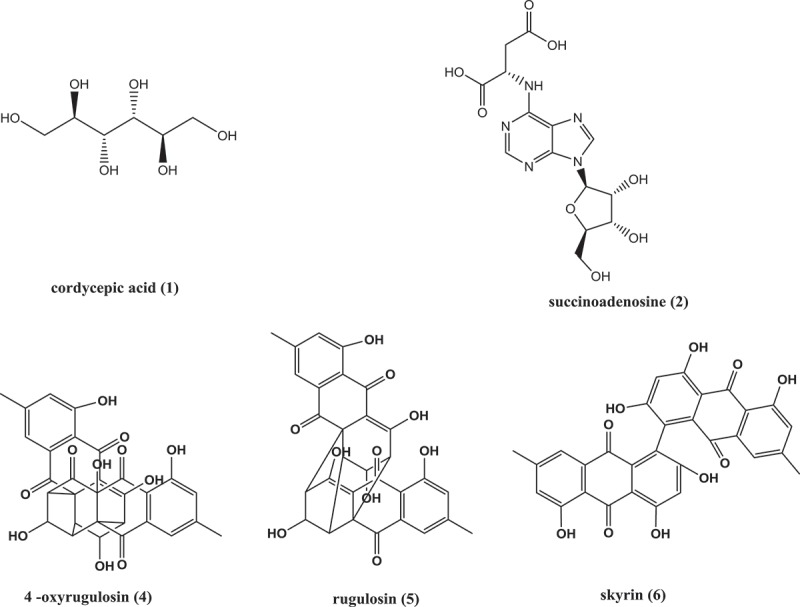
The structure of the five compounds.

Active components were collected at 13–14 min corresponding to the chromatographic peak at the retention time of 13.9 min shown in Figure 4. Mass spectra results are shown in Figure 8, which confirmed that in the anionic mode, it had a unique mass-to-charge ratio of 382.09880 (the relative error of 4.18 ppm), an excimer ion peak of [M − H]^−^ and the corresponding ionic formula was C_14_H_16_N_5_O_8_^−^. In the cationic mode, there is a peak with a mass-to-charge ratio (*m*/*z*) of 384.11493 (the relative error of 0.14 ppm), which is an excimer ion peak of [M + H]^+^, the corresponding ionic formula was C_14_H_18_N_5_O_8_^+^. According to the anion and cations analysis, the molecular formula of the component was C_14_H_17_N_5_O_8_. In combination with the natural products of related entomopathogenic fungi and the Chapman & Hall natural product database, it could be deduced that the active component is succinoadenosine shown in Figure 13.

Collected active components at 15–16 min corresponding to the chromatographic peak at the retention time of 16.1 min are shown in Figure 4, MS test results showed that the anionic mode had a unique mass-to-charge ratio of 486.21982 (the relative error is 1.5 ppm) (Figure 9), and it can be inferred that the corresponding ionic formula was C_20_H_32_N_5_O_9_^−^. In the cationic mode, there is a peak with a mass-to-charge ratio (*m*/*z*) of 488.23526 (the relative error of 0.33 ppm), which is an excimer ion peak of [M + H]^+^, and the corresponding ionic formula was C_20_H_34_N_5_O_9_^+^. According to the anion and cations analysis, the molecular formula of the component was C_20_H_33_N_5_O_9_. It was found that it had a maximum absorption peak at 258 nm from the chromatographic data. In combination with the natural products of related entomopathogenic fungi, and query the Chapman & Hall natural product database, we did not find a compound that matched it; it was initially deduced that the component might be a new compound.

##### 3.3.2.2. HRMS analysis of active components in methanol extracts

The active fraction at 3 min collected from the methanol extract in Figure 6, corresponding to Figure 5, there are two UV absorption peaks. The peaks were collected and tested for activity, and the activity was determined to confirm the active fraction as a chromatographic peak with a retention time of 3.1 min. Further analysis by MS showed that the compound was the same compound as the active ingredient in the aqueous extract with a retention time of 3.1 min, i.e. cordycepic acid.

The chromatographic peak retention time for the active fraction collected at 30 min in Figure 6 was 30.1 min, and the mass spectrum was obtained by MS according to the method 2.8 (Figure 10). It was found that the mass fraction ratio obtained in the anionic mode was 557.10753 with the relative error of 2.51 ppm, the excimer ion peak [M − H]^−^ and the corresponding ion type is C_30_H_21_O_11_^−^. In the cationic mode, there is a peak with a mass-to-charge ratio (*m*/*z*) of 559.12253 with the relative error of 1.71 ppm, which is an excimer ion peak of [M + H]^+^, and the corresponding ionic formula is C_30_H_23_O_11_^+^. According to the analysis results of anion and cations, it is concluded that the molecular formula of the component is C_30_H_22_O_11_. Combining with its UV absorption peak at 375 nm, consulting the relevant active ingredients reported, and querying Chapman & Hall natural product database, it was inferred that the component was 4α-oxyrugulosin, and the structural formula was shown in Figure 13.

The chromatographic peak retention time for the active fraction collected at 31 min in Figure 6 was 31.7 min, and the mass spectrum was obtained by MS (Figure 11). It was found that the mass fraction ratio obtained in the anionic mode was 541.11285 peak; from the ESI characteristics, it can be inferred that it is [M − H]^−^ the excimer ion peak, the corresponding ion type is C_30_H_21_O_10_^−^, the relative error of 2.16 ppm. In the cationic mode, there is a peak with a mass-to-charge ratio (*m*/*z*) of 543.12822, which is an excimer ion peak of [M + H]^+^, the corresponding ionic formula is C_30_H_23_O_10_^+^ and the relative error is 0.65 ppm. According to the analysis results of anion and cations, it concludes that the molecular formula of the component is C_30_H_22_O_10_. According to the chromatographic data, it was found that the UV absorption characteristic wavelength was 389 nm, and the Chapman & Hall natural product database was consulted and the results were discussed with reference to He’s antitumor component. It is deduced that the component is rugulosin (Figure 13).

Active components were collected at 34 min corresponding to the chromatographic peak at the retention time of 34.6 min shown in Figure 6 and the results of mass spectra shown in Figure 12. The results showed that the anionic mode has a unique peak at the mass-to-charge ratio of 537.08199 with the relative error of 1.36 ppm, which might be an excimer ion peak of [M − H]^−^ according to the ESI characteristic, and the corresponding ionic formula is C_30_H_17_O_10_^−^. In the cationic mode, there is a peak at the mass-to-charge ratio (*m*/*z*) of 539.09681 with the relative error of 0.86 ppm, which is an excimer ion peak of [M + H]^+^, and the corresponding ionic formula is C_30_H_19_O_10_^+^. According to the analysis results of anion and cations, it concludes that the molecular formula of the component is C_30_H_18_O_10_. According to the chromatographic data, the UV absorption characteristic wavelength was found at 453 nm, and the results were discussed with reference to He’s natural pigment after consulting Chapman & Hall natural product database. It was inferred that the component was skyrin (Figure 13).

## 4. Conclusion and discussion

In this study, the active components were analysed by liquid chromatography and high-resolution TOI MS (ESI-MS), and the molecular formulas and chemical structures of the active constituents were determined. Cordycepic acid is one of the main active ingredients of *Cordyceps*, which can be used to inhibit the growth of a variety of bacteria, regulate blood sugar and prevent cancer, cerebral thrombosis etc. (Holliday and Cleaver 2008). It is generally considered that the high content of cordycepic acid in *Cordyceps* indicates potentially important medicinal value.

According to the Chapman & Hall natural product database, it was found that the compound succinoadenosine was a metabolite of *Penicillium chrysogenum* and other microbes, and DPPH-free radical scavenging activity was not published previously in insect fungi.

Compound with the formula C_20_H_33_N_5_O_9_ has an unsaturation degree of 7 exhibited a strong polarity, depending on their scavenging activity on DPPH-free radicals and reference to the structural type of the parasite metabolite (Hu and Li 2007; Gaikwad et al. 2010), suggesting that the compound may be phenolic hydroxyl peptide, and the specific chemical structure remains to be further confirmed.

Compound 4α-oxyrugulosin, a metabolite of *Penicillium brunneum* (Shibata 1973), which has not been reported for its DPPH and hydroxyl radical scavenging activity, is also found for the first time in insect-borne fungi.

Rugulosin and skyrin have been reported as metabolites of *H*. *huangshanensis*, in which rugulosin has CHO cytotoxicity and skyrin does not exhibit CHO cytotoxicity (He et al. 2007, 2008). We found that rugulosin showed both DPPH-free radical and hydroxyl radical scavenging activity, while skyrin only exhibited DPPH-free radical scavenging activity.

The removal of free radicals or the blocking of the associated oxidation plays a major role in the life of the organism. The content of cordycepic acid was higher in the FMOF. Also, its metabolite rugulosin not only had CHO cytotoxicity but also showed DPPH and hydroxyl-free radical scavenging activity; 4α-oxyrugulosin with DPPH and hydroxyl-free radical scavenging activity was found for the first time in this fungus. It suggests that the active ingredient of mycelia from the fungus has potentially high value and deserves further research and development (Wang et al. 2015).
